# Neutralizing antibody response in the patients with hand, foot and mouth disease to enterovirus 71 and its clinical implications

**DOI:** 10.1186/1743-422X-8-306

**Published:** 2011-06-16

**Authors:** Chunfu Yang, Chaoyang Deng, Junfeng Wan, Liye Zhu, Qibin Leng

**Affiliations:** 1 Key Laboratory of Molecular Virology and Immunology, Institut Pasteur of Shanghai, Chinese Academy of Sciences, 225 South Chongqing Road, Shanghai, 200025, P.R. China; 2 Center for Disease Control of Fuyang city, 19 Zhongnan Avenue, Fuyang City, Anhui Province, 236300, P.R. China

**Keywords:** EV71, neutralizing antibody, hand, foot and mouth disease, cellular immune response, vaccine

## Abstract

Enterovirus 71 (EV71) has emerged as a significant pathogen causing large outbreaks in China for the past 3 years. Developing an EV71 vaccine is urgently needed to stop the spread of the disease; however, the adaptive immune response of humans to EV71 infection remains unclear. We examined the neutralizing antibody titers in HFMD patients and compared them to those of asymptomatic healthy children and young adults. We found that 80% of HFMD patients became positive for neutralizing antibodies against EV71 (GMT = 24.3) one day after the onset of illness. The antibody titers in the patients peaked two days (GMT = 79.5) after the illness appeared and were comparable to the level of adults (GMT = 45.2). Noticeably, the antibody response was not correlated with disease severity, suggesting that cellular immune response, besides neutralizing antibodies, could play critical role in controlling the outcome of EV71 infection in humans.

## Background

Historically, the outbreaks of HFMD (hand, foot and mouth disease) in European countries and the United States have been spontaneous and small in scale. Noticeably, there were also two large outbreaks with high mortality rates in Bulgaria (1975) [1] and Hungary (1978) [2]. In recent years numerous large outbreaks of HFMD have occurred in eastern and southeastern Asian countries and regions, including Malaysia [3], Singapore [4], Taiwan [5], Japan [6], South Korea [7], Vietnam [8] and mainland China [9]. HFMD has become an emerging disease in China since March 2008 [10]. Accumulating cases so far have reached 3.4 million, including nearly 1400 fatalities. It is worth mentioning that not only deaths have increased 156% over the last year, but the overall number of severe cases has also increased significantly. The noticeable difference of these outbreaks in China from other regions is that the circulating EV71 viruses are only from the C4 genotype [11], but the reasons for causing large outbreaks in China still remains largely unclear. Environmental, human genetic and immunological factors all have, most likely, contributed to it.

In recent years, several EV71 vaccine candidates, including live-attenuated virus, inactivated whole virus, recombinant viral protein, virus-like particle and DNA vaccine, have been evaluated in animal studies [12-17]. The vaccine studies in animal models have demonstrated that neutralizing antibodies may play a critical role in protecting mice from the viral challenge [12,13,16]. EV71 vaccine clinical trials have been approved recently and will be soon carried out in China. Other than the target populations, it is difficult to predict what antibody titer will be considered as a protective level in the clinical trials. Probably the best way to discover this information is to study naturally-occurring EV71 infections in patients.

Because the current outbreak in China began in March 2008 in the city of Fuyang, located in northwestern Anhui province [18], we retrospectively compared the antibody response of patients to EV71 infection in Fuyang to asymptomatic healthy children and young adults. We found that neutralizing antibody titers against EV71 was detectable one day after symptoms manifested and reached peak levels 2 days afterwards. To our surprise, the antibody response was not correlated with severity of the disease. In addition, more than 17% patients had less than a 1:16 neutralizing antibody titer. Thus, our data suggests that the neutralizing antibody may not be the only mechanisms protecting adults as well as children from EV71 infection.

## Results

In 2008, there were 7,470 reported cases of HFMD in the city of Fuyang during the period from March 1 to May 31, of which 4840 patients were hospitalized and 23 cases were fatal (case fatality rate = 0.31%). The number of cases, according to date of onset, began to increase in early April and peaked on April 28. The number of reported HFMD cases in Fuyang decreased after May 5. RT-PCR analysis of EV71 nucleic acid revealed that 69 of 161 cases (60.3%) were positive in various specimens, including pharyngeal swabs, lung puncture fluid, lung tissues and blood. All of them were negative in RT-PCR analysis of Coxsackievirus A16 (CA16) nucleic acid. Therefore, EV71 but not CA16 was determined to be the causative pathogen.

In this study, we analyzed 58 HFMD patients and 60 healthy controls. Both groups ranged in age from 1 to 3 years old. Among the patients, 13 of them had typical HFMD symptoms such as a skin rash and fever and were assigned to the mild group. Twenty-eight patients were critical with severe neurological pulmonary edema. Seventeen had less CNS involvement such as poliomyelitis-like syndrome, acute flaccid paralysis, encephalomyelitis, aseptic meningitis or encephalitis.

**Figure 1:**
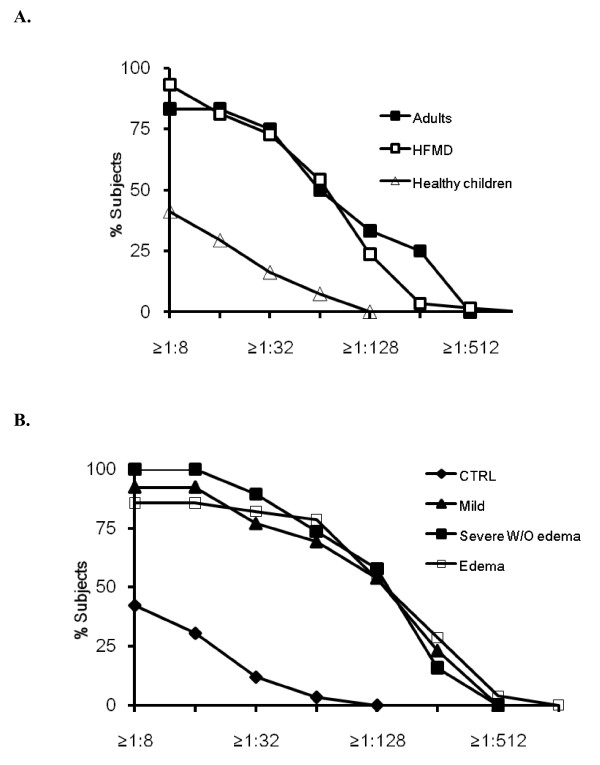
**Reverse cumulative distribution curves of neutralizing antibody titers in HFMD patients, healthy children and adults subjects by microneutralization assay against EV71**. A. HFMD patients were analyzed as one group. B. HFMD patients were further categorized into mild group, severe group without pulmonary edema and severe group with pulmonary edema in accordance with their disease severity.

Similar to previous finding [10], we found that 41.2% of HFMD-asymptomatic healthy children had neutralizing antibody titers of 1:8 or above against EV71 (geometric mean titer, GMT = 7.4). The proportion of the healthy controls that had neutralizing antibody titers ≥ 1:16 and ≥ 1:64 dropped to 30.6% and 3.4%, respectively, while none of them had ≥ 1:128 antibody titers. In contrast, 10 of 12 adults (83.3%) had neutralizing antibody titers ≥ 1:16, which were significantly higher (GMT = 45.3) than those of the healthy children. Of note, 4 of 12 adults had neutralizing antibody titers ≥ 1:128 (Figure 1A).

Overall HFMD patients had remarkably higher levels of neutralizing antibody titers (GMT = 72) than the healthy controls (p < 0.01). The proportion of patients having neutralizing antibody titers ≥ 1:8, ≥ 1:16, ≥ 1:32, ≥ 1:64, ≥ 1:128, ≥ 1:256, ≥ 1:512 were 93.2%, 81.3%, 72.9%, 54.2%, 23.7%, 3.4% and 1.7%, respectively. The antibody titers in the patients with CNS involvement or pulmonary edema were not significantly different from those of patients with mild HFMD [Figure 1B], suggesting that the neutralizing antibody titers were not associated with the severity of the disease.

**Figure 2:**
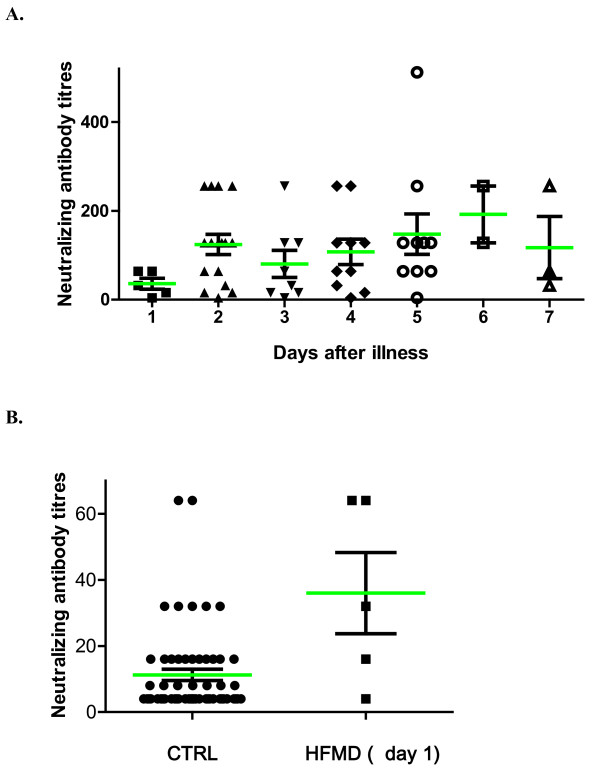
**Neutralizing antibody titers in HFMD patients by microneutralization assay against EV71**. A. HFMD patients were classified by dates of onset of illness. B. Serum samples of HFMD patients taken one day after symptoms of illness appeared had significantly higher antibody titers than control subjects (p < 0.05).

Because the serum samples were collected at various periods after the appearance of symptoms, we questioned whether the antibody titers were associated with the collection times. Overall, our analysis revealed that the antibody titers were not correlated with the collection times. Noticeably, the serum samples collected one day after the onset of illness had significantly lower levels of neutralizing antibodies than the samples collected at a later time [Figure 2A]. However, the neutralizing antibody titers from the serum samples of patients collected one day after falling ill (GMT = 24.3) were already more than 3 times higher than that of healthy control children (GMT = 7.4) [Figure 2B]. In addition, 4 of 5 patients had neutralizing antibody titers above 1:16. These data suggest that neutralizing antibodies are typically produced when symptoms appear in patients.

## Methods and materials

### Human subjects and serum samples

The enrollment of HFMD patients was reported by CDC to the WHO previously [18]. Accordingly, all the patients had the following symptoms: 1) skin rash or blisters on hand, foot, or buttock, and fever, in the absence of measles, rubella, chicken pox and other febrile eruption diseases; 2) skin rash or blisters on hand, foot, or buttock, and ulcers on the mouth or mucous membrane, in the absence of drug-related rash or allergy. Severe cases were defined as having two of following clinical manifestations: 1) continuous high fever, 2) weakness, vomiting, irritability, etc., 3) abnormal white blood cell count, 4) high blood glucose level or/and 5) poor blood circulation of limbs. Pulmonary edema was defined as respiratory distress, associated with tachycardia, tachypnea, rales, and copious frothy sputum, with a chest roentogenograph that showed bilateral pulmonary infiltrates without cardiomegaly [19].

Serum samples from control children, adults and HFMD patients were collected by the Center for Disease Control of Fuyang city for the purpose of national HFMD surveillance in 2008 [18]. Written informed consents were obtained for the use of serum samples from all participants (or their parents). This study has been approved by the ethics committee of Center for Disease Control of Fuyang city. The serum samples from the patients whose ages ranged from 1-3 years old were analyzed in this study.

### Neutralization assay

The methods for measuring EV71 neutralizing antibodies on microtiter plates have been described previously [20-22]. Briefly, heat-inactivated (56°C for 30 min) sera were serially diluted in two-fold steps in Eagle's minimal essential medium containing 2% fetal bovine serum (MEM-2), with an initial serum dilution of 1:8. The tissue culture infectious dose (TCID_50_) of the viral stocks was determined and then diluted 10-fold, 10^2^-fold and 10^3^-fold with MEM-2. Diluted sera and 100 TCID_50 _of viral samples were combined (50 μl of each) and the mixture was incubated at 37°C for 1 hour. The virus-serum mixtures (total 200 μl) were then inoculated on 70%-confluent rhabdomyosarcoma cells in a 96-well plate and monitored for the development of characteristic cytopathic effects for 4 days. The neutralizing titer of a particular serum and virus was defined as the reciprocal of 100% of the highest dilution that resulted in no observable cytopathic effect.

The EV71 FY573 isolate (subgenotype C4a, GenBank accession number: HM064456.1) used in this study was isolated from a patient with HFMD from Fuyang city of Anhui province in 2008.

### Statistics

GraphPad Prism 5 was used in statistic analysis and graph preparations. P-values were calculated by student T-test.

## Discussion

Studies of animal models revealed that antibody response plays a critical role in protection from EV71 infection [12-16]. Several seroepidemiological studies in humans have revealed that seroprevalence of EV71 antibodies increase with age [10,23,24], which supports the idea that increased humoral immunity may prevent adults from EV71 infection. Thus, it is of importance to know how humans respond to EV71 infection and to what degree humoral immune responses play a role in EV71 infection. We found that 23.7% of HFMD patients had ≥ 1:128 antibody titers, but none of the healthy children did. In addition, we found that 80% (4/5) of patients had neutralizing antibody titers against EV71 above 1:16 one day after the onset of illness. Two days after symptoms appeared, patients already had a level of neutralizing antibodies (GMT = 79.5) comparable to that of adults. In other words, humoral immune response was already elicited when clinical symptoms appeared. Interestingly, our data is in accordance with a previous report showing that neutralizing antibody levels are not correlated with HFMD severity [25]. However, this current study was a retrospective investigation and we only had a very small number of cases at early stages of infection. Whether the neutralizing antibody responses in patients to EV71 at early stages of infection correlate to the clinical severity of the disease remains to be determined by future investigations.

Previously Gomes et al. showed that only 40.8% of HFMD patients less than 3 years old had levels of neutralizing antibody ≥ 1:8 [26]. Their results are not consistent with the results in this study or with the results reported by Chang et al [25]. This discrepancy may result from technical problems by using different EV71 isolates. They used classic EV71 strains (BrCr) but not a contemporary circulating EV71 isolate. In addition, the pathogens causing HFMD in the Brazilian study may not be only EV71 since they did not specify in the study.

Recently, a clinical trial for an EV71 vaccine has been officially approved in China (http://clinicaltrials.gov/ct2/show/NCT01267903). While there has been no clinical trial thus far for the EV71 vaccine, it is imperative to know what level of neutralizing antibody should be achieved in children after EV71 vaccination and the importance of cellular immunity in protection from EV71 infection. Adults are rarely infected with EV71 [27], thus they appear to have immunity against EV71 infection. Only 83.3% of healthy adults in this study were antibody-positive to EV71 (≥ 1:8 antibody titer). Even HFMD patients were not all positive (93.2%). In addition, 18.7% of HFMD patients did not have antibody titers ≥ 1:16. Furthermore, similar to a previous study by Chang et al., we found that there was no correlation between the severity of the disease and neutralizing antibody levels. Thus, one may wonder, other than neutralizing antibodies, additional immune mechanisms may also contribute to the immunity against EV71 infection in humans.

Interestingly, both human leukocyte antigen serotypes, HLA-A33 (class I) and HLA-DR17 (class II), are significantly associated with EV71 infection, suggesting cellular immune responses may play an important role in human immunity against EV71 infection [28]. In addition, a previous study found that the most severe EV71 cases, characterized by with pulmonary edema, had lower cellular antigen-specific Th1 cytokines coupled with a lower lymphocyte proliferation response to EV71 antigen in comparison to milder cases, which suggests that cellular immune responses are correlated to the clinical severity of HFMD [25]. Thus, induction of both humoral and cellular immunity should be considered in vaccine development and further analyzed by clinical trials.

## List of abbreviations

EV71: Enterovirus 71; HFMD: Hand, foot and mouth disease; GMT: geometric mean titer; TCID: tissue culture infectious dose.

## Competing interests

The authors declare that they have no competing interests.

## Authors' contributions

CY performed the experiments. LZ and JW performed the field study. CY and LQ designed the study, analyzed the data and wrote this manuscript. All authors read and approved the final manuscript.

## References

[B1] Chumakov M Voroshilova M Shindarov L Lavrova I Gracheva L Koroleva G Vasilenko S Brodvarova I Nikolova M Gyurova S Enterovirus 71 isolated from cases of epidemic poliomyelitis-like disease in Bulgaria Arch Virol 1979 60 329 340 10.1007/BF01317504 228639

[B2] Nagy G Takatsy S Kukan E Mihaly I Domok I Virological diagnosis of enterovirus type 71 infections: experiences gained during an epidemic of acute CNS diseases in Hungary in 1978 Arch Virol 1982 71 217 227 10.1007/BF01314873 6285858

[B3] Chan LG Parashar UD Lye MS Ong FG Zaki SR Alexander JP Ho KK Han LL Pallansch MA Suleiman AB Deaths of children during an outbreak of hand, foot, and mouth disease in sarawak, malaysia: clinical and pathological characteristics of the disease. For the Outbreak Study Group Clin Infect Dis 2000 31 678 683 10.1086/314032 11017815

[B4] Chong CY Chan KP Shah VA Ng WY Lau G Teo TE Lai SH Ling AE Hand, foot and mouth disease in Singapore: a comparison of fatal and non-fatal cases Acta Paediatr 2003 92 1163 1169 10.1111/j.1651-2227.2003.tb02478.x 14632332

[B5] Ho M Chen ER Hsu KH Twu SJ Chen KT Tsai SF Wang JR Shih SR An epidemic of enterovirus 71 infection in Taiwan. Taiwan Enterovirus Epidemic Working Group N Engl J Med 1999 341 929 935 10.1056/NEJM199909233411301 10498487

[B6] Fujimoto T Chikahira M Yoshida S Ebira H Hasegawa A Totsuka A Nishio O Outbreak of central nervous system disease associated with hand, foot, and mouth disease in Japan during the summer of 2000: detection and molecular epidemiology of enterovirus 71 Microbiol Immunol 2002 46 621 627 12437029 10.1111/j.1348-0421.2002.tb02743.x

[B7] Jee YM Cheon DS Kim K Cho JH Chung YS Lee J Lee SH Park KS Lee JH Kim EC Genetic analysis of the VP1 region of human enterovirus 71 strains isolated in Korea during 2000 Arch Virol 2003 148 1735 1746 10.1007/s00705-003-0133-6 14505086

[B8] Tu PV Thao NT Perera D Huu TK Tien NT Thuong TC How OM Cardosa MJ McMinn PC Epidemiologic and virologic investigation of hand, foot, and mouth disease, southern Vietnam, 2005 Emerg Infect Dis 2007 13 1733 1741 18217559 10.3201/eid1311.070632 PMC3375788

[B9] Zhang Y Tan XJ Wang HY Yan DM Zhu SL Wang DY Ji F Wang XJ Gao YJ Chen L An outbreak of hand, foot, and mouth disease associated with subgenotype C4 of human enterovirus 71 in Shandong, China J Clin Virol 2009 44 262 267 10.1016/j.jcv.2009.02.002 19269888

[B10] Zhu Z Zhu S Guo X Wang J Wang D Yan D Tan X Tang L Zhu H Yang Z Retrospective seroepidemiology indicated that human enterovirus 71 and coxsackievirus A16 circulated wildly in central and southern China before large-scale outbreaks from 2008 Virol J 2010 7 300 10.1186/1743-422X-7-300 21050463 PMC2989968

[B11] Wang LC Tang SQ Li YM Zhao HL Dong CH Cui PF Ma SH Liao Y Liu LD Li QH A comparison of the biological characteristics of EV71 C4 subtypes from different epidemic strains Virol Sin 2010 25 98 106 10.1007/s12250-010-3102-8 20960306 PMC8227828

[B12] Chen HL Huang JY Chu TW Tsai TC Hung CM Lin CC Liu FC Wang LC Chen YJ Lin MF Chen CM Expression of VP1 protein in the milk of transgenic mice: a potential oral vaccine protects against enterovirus 71 infection Vaccine 2008 26 2882 2889 10.1016/j.vaccine.2008.03.041 18450335

[B13] Chung CY Chen CY Lin SY Chung YC Chiu HY Chi WK Lin YL Chiang BL Chen WJ Hu YC Enterovirus 71 virus-like particle vaccine: improved production conditions for enhanced yield Vaccine 2010 28 6951 6957 10.1016/j.vaccine.2010.08.052 20797455

[B14] Ong KC Devi S Cardosa MJ Wong KT Formaldehyde-inactivated whole-virus vaccine protects a murine model of enterovirus 71 encephalomyelitis against disease J Virol 2010 84 661 665 10.1128/JVI.00999-09 19864378 PMC2798416

[B15] Xu J Qian Y Wang S Serrano JM Li W Huang Z Lu S EV71: an emerging infectious disease vaccine target in the Far East? Vaccine 2010 28 3516 3521 10.1016/j.vaccine.2010.03.003 20304038

[B16] Zhang D Lu J Enterovirus 71 vaccine: close but still far Int J Infect Dis 2010 14 e739 743 10.1016/j.ijid.2009.12.002 20400350 PMC7110504

[B17] Arita M Shimizu H Nagata N Ami Y Suzaki Y Sata T Iwasaki T Miyamura T Temperature-sensitive mutants of enterovirus 71 show attenuation in cynomolgus monkeys J Gen Virol 2005 86 1391 1401 10.1099/vir.0.80784-0 15831951

[B18] WHO Report on the Hand, Foot and Mouth Disease Outbreak in Fuyang City, Anhui Province and the Prevention and Control in China 2008 1 25

[B19] Wang SM Lei HY Huang MC Wu JM Chen CT Wang JN Wang JR Liu CC Therapeutic efficacy of milrinone in the management of enterovirus 71-induced pulmonary edema Pediatr Pulmonol 2005 39 219 223 10.1002/ppul.20157 15635619

[B20] Ooi EE Phoon MC Ishak B Chan SH Seroepidemiology of human enterovirus 71, Singapore Emerg Infect Dis 2002 8 995 997 12194783 10.3201/eid0809.10.3201/eid0809.010397 PMC2732542

[B21] Cao R Han J Deng Y Yu M Qin E Qin C Presence of high-titer neutralizing antibodies against enterovirus 71 in intravenous immunoglobulin manufactured from Chinese donors Clin Infect Dis 2010 50 125 126 20001529 10.1086/649012

[B22] Huang ML Chiang PS Luo ST Liou GY Lee MS Development of a high-throughput assay for measuring serum neutralizing antibody against enterovirus 71 J Virol Methods 2010 165 42 45 10.1016/j.jviromet.2009.12.015 20036286

[B23] Diedrich S Weinbrecht A Schreier E Seroprevalence and molecular epidemiology of enterovirus 71 in Germany Arch Virol 2009 154 1139 1142 10.1007/s00705-009-0413-x 19506798

[B24] Rabenau HF Richter M Doerr HW Hand, foot and mouth disease: seroprevalence of Coxsackie A16 and Enterovirus 71 in Germany Med Microbiol Immunol 2010 199 45 51 10.1007/s00430-009-0133-6 19941005

[B25] Chang LY Hsiung CA Lu CY Lin TY Huang FY Lai YH Chiang YP Chiang BL Lee CY Huang LM Status of cellular rather than humoral immunity is correlated with clinical outcome of enterovirus 71 Pediatr Res 2006 60 466 471 10.1203/01.pdr.0000238247.86041.19 16940249 PMC7086547

[B26] Gomes Mde L de Castro CM Oliveira MJ da Silva EE Neutralizing antibodies to enterovirus 71 in Belem, Brazil Mem Inst Oswaldo Cruz 2002 97 47 49 11992146 10.1590/s0074-02762002000100006

[B27] Hamaguchi T Fujisawa H Sakai K Okino S Kurosaki N Nishimura Y Shimizu H Yamada M Acute encephalitis caused by intrafamilial transmission of enterovirus 71 in adult Emerg Infect Dis 2008 14 828 830 10.3201/eid1405.071121 18439374 PMC2600258

[B28] Chang LY Chang IS Chen WJ Huang YC Chen GW Shih SR Juang JL Shih HM Hsiung CA Lin TY Huang LM HLA-A33 is associated with susceptibility to enterovirus 71 infection Pediatrics 2008 122 1271 1276 10.1542/peds.2007-3735 19047245

